# Safety and Efficacy of Tianfoshen Oral Liquid in Non-Small Cell Lung Cancer Patients as an Adjuvant Therapy

**DOI:** 10.1155/2019/1375439

**Published:** 2019-04-03

**Authors:** Lei Zhao, Jing Wang, Huihui Li, Juanjuan Che, Nina Ma, Bangwei Cao

**Affiliations:** Cancer Center, Beijing Friendship Hospital, Capital Medical University, Beijing 100050, China

## Abstract

**Ethnopharmacological Relevance:**

As an important Chinese herb injection, Tianfoshen (TFS) oral liquid is widely used in Chinese non-small cell lung cancer (NSCLC) patients.

**Aim of the Study:**

To evaluate the efficacy and safety of Tianfoshen (TFS) oral liquid plus chemotherapy in Chinese NSCLC patients with Qi and Yin deficiency syndrome, an observational study was conducted in Beijing Friendship Hospital between August 2012 and July 2016. Patients, enrolled in this study, were diagnosed with NSCLC and were treated with Cisplatin in combination with Paclitaxel/Navelbine/Gemcitabine/Docetaxel as a first-line treatment, or Pemetrexed for recurrent patients. The primary endpoint was the improvement of traditional Chinese medicine syndrome and objective response rate in patients. The secondary endpoint was the occurrence of drug-related adverse events.

**Results:**

A total of 108 patients were included in this study and underwent the safety and efficacy assessments. Compared to the baseline, the total scores of traditional Chinese medicine (TCM) syndrome after 3 or 8/9 weeks of TFS treatment were statistically significant (*P*<0.0001), and the clinical efficiency rate was 36.11% and 50.93%, respectively. The objective response rate (ORR) and disease control rate (DCR) of TFS group were slightly higher than those of without-TFS group, although the comparison was not statistically significant. The incidence of common adverse events related to TFS was 7.41% whereas the incidence of serious adverse events was 0.93%.

**Conclusions:**

As an adjuvant therapy of chemotherapy, TFS showed an acceptable tolerability profile in the clinical practice of Chinese NSCLC patients with Qi and Yin deficiency syndrome, but it seems to have no effect on the ORR and DCR.

## 1. Introduction

Lung cancer is one of the most common malignancies with 1.8 million newly diagnosed cases (about 13% of the total cancer diagnoses) and almost 1.6 million estimated deaths every year [1]. About 85% of the lung cancer patients are non-small cell lung cancer (NSCLC). Most NSCLC patients were diagnosed at an advanced stage where surgical intervention is not applicable and only chemotherapy and radiotherapy are the main therapeutic approaches for these patients [2]. However, almost all chemotherapies are associated with severe side effects and usually result in a decreased life quality for NSCLC patients [3]. Novel therapeutic strategies are necessary to improve the clinical outcomes with fewer side effects for patients with NSCLC.

The broad application of the complementary and alternative medicine (CAM) has grabbed global attention, especially for cancer prevention and treatment. Traditional Chinese medicine (TCM) is one of the most important types of CAM, with a unique system of diagnostics and therapies that rely on the accumulative clinical experience gained and translated through generations of doctors. Chinese herbal medicines, which contain multiple biologically active components with pleiotropic therapeutic efficacies, have minimal side effects and provide exclusive sources for the development of new drugs [3].

Tianfoshen (TFS) oral liquid (Changshu Leiyunshang Pharmaceutical Co., Ltd., Su Zhou, China, Z20080051) is one of the highly active Chinese herbal medicine prescriptions and is mainly composed of lucid asparagus, fructus citri sarcodactylis, American ginseng, Acanthopanax Obovatus, Chinese Actinidia root, and the dried toad venom [4]. TFS oral liquid received a certificate as an authorized new drug in September 2008 and hence its clinical application started. Studies have suggested that TFS oral liquid could improve the quality of life and the immune function of liver cancer and stomach cancer patients in clinical studies [5–7]. Clinical studies also showed that TFS could improve symptoms, living quality, and the immune function of NSCLC patients with Qi and Yin deficiency syndrome [5–7]. TFS induced apoptosis in HEp-2 human laryngeal cancer cells [8] and was able to reverse the drug resistance of human erythrocyte leukemic cell K562/ADM [8]. Moreover, TFS was found to be lethal in cancer cells and also inhibited the proliferation of single clonogenic cells, with a broad-spectrum dose-dependent inhibitory action on various tumors [9]. However, the previous clinical studies on TFS oral liquid were conducted with a small sample size; thus clinical studies with large sample size are necessary to prove the clinical efficacy and safety of this TFS.

Since TFS is an investigational new drug (state medical permit no. Z20080051), the efficacy and toxicity profile can be different according to the population and ethnic differences, so further assessment of its efficacy and any side effects in more patients is requested. This study was designed to evaluate the efficacy and safety of TFS plus chemotherapy in Chinese NSCLC patients with advanced Qi and Yin deficiency syndrome.

## 2. Methods

### 2.1. Patient Eligibility

Patients with NSCLC, diagnosed by histological or cytological examinations, were eligible for this study. The other key inclusion criteria include the following: patients have Eastern Cooperative Oncology Group (ECOG) performance status of 0-2; patients should have adequate hematological, hepatic, and renal function; traditional Chinese medicine syndrome differentiation is according to the standard of Qi and Yin deficiency syndrome; patients should have expected overall survival time of more than 3 months and be from 18 to 75 years old.

The key exclusion criteria include the following: patients who had participated in other clinical trials within 3 months before enrollment, are allergic to the TFS or its constitution, or taking digitalis; patients who are psychopath, pregnant, or lactating mothers.

### 2.2. Study Design

This study was conducted in accordance with the Declaration of Helsinki of 1975 (revised in 2000) and approved by an independent ethics committee in Beijing Friendship Hospital. All patients provided written informed consent for the administration of the tested drug and the authorization to publish this paper.

This study was intended to evaluate the efficacy and safety of TFS plus chemotherapy in Chinese NSCLC patients with advanced Qi and Yin deficiency syndrome. The objective response rate (ORR) and disease control rate (DCR) data of patients who received the same chemotherapy regimen with and without TFS were analyzed, and hierarchical analysis was conducted according to first-line treatment and recurrent therapy. The production quality of TFS in this study is in accordance with the relevant requirements of* the Standard for Quality Management of Chinese Medicinal Materials (GAP)* (2002).

The Qi and Yin deficiency syndrome was diagnosed according to the diagnostic criteria *⟪*Guidelines for Clinical Research of Traditional Chinese Medicine*⟫* published in 2002. The main symptoms are cough, sputum less, sticky sputum, bloody sputum, cough sound low, and shortness of breath. The secondary symptoms are fatigue, pale, being disgusted of the wind, spontaneous or night sweats, dry mouth, and less drinking. The tongue pulses are red or the tongue is pink, with thin fur and thin weak pulse. Patients with two main symptoms, or two secondary symptoms and one main symptom, or more than three secondary symptoms and tongue pulse can be defined as Qi and Yin deficiency syndrome.

### 2.3. Treatment

Advanced NSCLC patients with first-line treatment or postoperative adjuvant therapy received a maximum of three cycles of intravenous Cisplatin (75 mg/m^2^) with Paclitaxel (175mg/m^2^)/Navelbine (25mg/m^2^)/Gemcitabine (1000mg/m^2^)/Docetaxel (75mg/m^2^) chemotherapy (administered on days 1-3, every 3 weeks). The recurrent patients received intravenous monotherapy of Pemetrexed (500mg/m^2^), administered on day 1 and then every 3 weeks. Patients started administration of TFS on the first day of chemotherapy. If the patient needed only 2 cycles of chemotherapy (6 weeks), then TFS was continued for two more weeks after the completion of chemotherapy (a total of 8 weeks for TFS application); if the patient needed to complete 3 cycles of chemotherapy, TFS was given for the same duration as chemotherapy (a total of 9 weeks for TFS application); then the safety and efficacy were assessed.

 Patients who withdrew due to intolerance of TFS-related or chemotherapy-related toxicity or disease deterioration needed to be immediately transferred to emergency treatment; if patients were treated less than three weeks, then only the safety was assessed. 

### 2.4. Toxicity Evaluation and Assessment of Response

The primary endpoint of this study was to assess the efficacy of TFS, evidenced by the improvement of patients in traditional Chinese medicine (TCM) syndrome and objective response rate. The secondary endpoint of this study was to assess the safety of TFS.

The profile of TCM syndromes mainly included cough, bloody phlegm, physical and mental fatigue, inappetence, dry throat, spontaneous perspiration, and night sweat. They were evaluated, respectively, by TCM syndrome diagnostic efficacy standards [9], at the time points of 0 days, 3 weeks, 8 or 9 (8/9) weeks after the treatment.

TCM syndrome score for the therapeutic effect was conducted according to the Nimodipine method [Efficacy index = (score before treatment - score after treatment) / score before treatment × 100%]. Clinical control of the patients means that almost all the symptoms disappeared after treatment (the efficacy index ≥90%); obvious therapeutic effect means that most symptoms disappeared (≥60% the efficacy index < 90%); ‘with therapeutic effect' means that the symptoms improved (≥30% the efficacy index < 60%); ‘without therapeutic effect' means that the symptoms had no obvious improving or worsening (the efficacy index < 30%). The clinical efficiency was calculated by the formula: clinical efficiency = (clinical control case number + obvious effective case number + effective number) / total case number × 100%.

Computed tomography (CT) scan was performed to assess tumor status, which was performed once within 4 weeks before the study registration and repeated once 9 weeks after the treatment. Response rates were evaluated by investigators according to Response Evaluation Criteria in Solid Tumors (RECIST, version 1.1) [10]. The ORR was defined as the sum of complete response (CR) and partial response (PR). The clinical benefit rate (CBR) was defined as the sum of CR, PR, and stable disease (SD). We compared the ORR and DCR data of patients who received the same chemotherapy regimen with and without TFS, and hierarchical analysis was conducted according to first-line treatment and recurrent therapy.

Weight gain was defined as the weight of patients after treatment increased ≥1kg versus the baseline; stable weight was defined as the weight of patients after treatment increased or decreased in less than 1 kg versus the baseline; weight loss was defined as the weight of patients after treatment decreased ≥ 1 kg versus the baseline.

Adverse events were assessed for all enrolled patients according to the Common Terminology Criteria for Adverse Events, version 4.0 [11].

### 2.5. Statistical Analysis

Data were analyzed with SPSS 20.0 statistical software (IBM SPSS Inc., Chicago, IL, USA). The primary hypotheses were evaluated with two-sided analysis. The mean, standard deviation, median, minimum, and maximum were used to present the quantitative data. Cases and percentage were used to present the categorical data. Quantitative data were compared by t-test or nonparametric rank sum test. The t-test was used to compare the differences before and after TFS treatment in TCM syndrome score, weight, and the subitems of Functional Assessment Of Cancer Therapy-Lung (FACT-L). The nonparametric rank sum test was used to compare the differences before and after TFS treatment in ECOG.* P* value less than 0.05 was considered as statistically significant.

## 3. Results

### 3.1. Patient Characteristics

A total of 115 patients were enrolled in the study between August 2012 and July 2016 in Beijing Friendship Hospital. After the exclusion of 7 patients due to noncompliance to follow-up, a total of 108 patients were included in this study. All patients were assessed for the safety and efficacy and were evaluated for cancer response. The demographic and baseline characteristics for the intention-to-treat (ITT) population were listed in Table 1. Patients enrolled in this study include 76 male and 32 female patients with a median age of 56 years (range 30-75). Distributions of the TMN staging in these patients were as follows: 22 patients (20.37%) were IIIB, 26 patients (24.07%) were IIIB, and 60 patients (55.56%) were IV. The distributions of the ECOG PS were 12 patients (11.11%) at 0, 82 patients (75.93%) at 1, and 14 patients (12.96%) at 2. The pathological diagnosis was 79 patients (73.15%) with adenocarcinoma, 23 patients (21.30%) with squamous carcinoma, and 6 patients (5.55%) with adenosquamous carcinoma. Affected organs included any organ in 75 patients (69.44%), the lung in 32 patients (29.63%), the liver in 6 patients (5.56%), and the lymph nodes in 22(20.37%) patients. The lung was the most common location of metastasis.

### 3.2. Efficacy

#### 3.2.1. ORR

A total of 95 patients treated with chemotherapy and TFS (TFS group) and 100 patients treated only with chemotherapy (without-TFS group) were included for the evaluation of cancer response. The ORR of patients with first-line treatment in the TFS group and without-TFS group was 34.21% and 30.95% (P=0.756). The ORR of patients with recurrent treatment in TFS group and without-TFS group was 26.42% and 22.41% (*P*=0.624). The disease clinical rate (DCR) of patients with first-line treatment in the TFS group and without-TFS group was 73.68% and 66.66% (P=0.494). The ORR of patients with recurrent treatment in TFS group and without-TFS group was 67.93% and 62.07% (*P*=0.519) (Table 2). Although the ORR and DCR of TFS group were slightly higher than those of without-TFS group, the comparison was not statistically significant.

#### 3.2.2. TCM Syndrome

The result of TCM syndrome for all patients is shown in Table 3. After 3 weeks of TFS treatment, a clinical control was observed in 2 patients, an obvious treatment effect was observed in 13 patients, and a mild treatment effect was observed in 29 patients. After 8/9 weeks of TFS treatment, a clinical control was observed in 4 patients, an obvious treatment effect was observed in 23 patients, and a mild treatment effect was observed in 45 patients. Overall, the clinical efficiency rate was increased from 40.74% (after 3 weeks of TFS treatment) to 66.67% (after 8/9 weeks of TFS treatment).

The mean TCM syndrome total scores of baseline, 3 weeks after TFS treatment, and 8/9 weeks after TFS treatment were 5.15 (2.20), 4.51 (2.35), and 3.84 (2.10), respectively. Compared with the baseline, TCM syndrome total score after 3 or 8/9 weeks of TFS treatment was statistically significant (*P*<0.0001, Table 4).

#### 3.2.3. Weight Changes

After 3 weeks and 8/9 weeks of TFS treatment, weight gain was found in 33 patients and 45 patients, stable weight in 53 patients and 36 patients, and weight loss in 22 patients and 27 patients, respectively. Compared to the baseline, weight score after 8/9 weeks of TFS treatment was statistically significant (*P*= 0.0023), whereas weight score after three weeks of TFS treatment showed no statistical significance (*P*>0.05, Table 4).

#### 3.2.4. ECOG

The results of signed rank sum test showed that after 3 weeks and 8/9 weeks of TFS treatment, the ECOG score has been greatly improved (*P*<0.05), compared to the baseline score (Table 2).

#### 3.2.5. Functional Assessment of Cancer Therapy-Lung (FACT-L)

All types of scores increased over time. Compared to the baseline score, the social/family score after 3 weeks of TFS treatment was statistically significant (*P*>0.05). However, the scores of physiology, emotion, function, pain, and additional attention, in both 3 weeks and 8/9 weeks of TFS treatment groups, were all statistically significant compared to those before treatment (*P*<0.05, Table 4).

### 3.3. Safety

During the entire study, the incidence of adverse events was 7.41% (8/108). No serious adverse events related to the tested drug were found. Chemotherapy toxicity appeared in 25 patients, which was related to chemotherapy drugs in 17 cases and related to TFS in 8 cases. Toxicities related to TFS are summarized in Table 4. Most of the toxicities were of grade 1 or 2. Frequent nonhematological toxicities including nausea/vomiting, fatigue, and proteinuria were observed (Table 5).

## 4. Discussion

Currently, chemotherapy is still one of the most important strategies of combination therapy in NSCLC [12]. A large number of clinical studies and meta-analyses showed that chemotherapy compared with best supportive treatment could significantly improve the survival and life quality of NSCLC patients [13]. The major adverse effects of chemotherapy regimens are bone marrow suppression, nephrotoxicity, and neurotoxicity. More cycles of chemotherapy can cause toxic accumulation and lead to other associated symptoms and signs, which makes appropriate chemotherapy treatment for NSCLC a very prominent problem. With the broad application of TCM in lung cancer treatment and the improvement of clinical effects, TCM showed a unique role in the comprehensive treatment of lung cancer. Meanwhile, TCM shows anticancer activity by promoting leucocytes and improving immune functions. It is mainly used as an adjuvant therapy with cancer chemotherapies to improve the quality of life (QOL) and prolong the survival period of cancer patients. Moreover, TCM maintenance treatment showed similar effects on time to progress (TTP) and overall survival (OS) compared to maintenance chemotherapy; however, it could improve QOL and increase 1-year survival rate of patients [14]. Another study showed that Chinese herbal decoction (CHD) could improve progression-free survival (PFS) and postprogression survival (PPS), which are closely related to treatment time and response degree of first-line treatment of small cell lung cancer (SCLC). CHD could improve body function and keep patients in a relatively stable condition [15].

An observational study was conducted in our center to assess the efficacy and safety of TFS as an adjuvant therapy to Chinese NSCLC patients with Qi and Yin deficiency syndrome. A total of 108 cases of NSCLC patients were included, and our results showed that TFS could significantly reduce the patient's TCM syndrome and ECOG and improve patient's weight and FACT-L. The most frequent nonhematological adverse event included nausea/vomiting, fatigue, and proteinuria. Most of the adverse events were of grade 1 or 2. This is the first time to systematically evaluate the Chinese medicine syndrome improvement by TFS concomitant administration with chemotherapy in a large sample population of NSCLC patients.

A clinical study containing 71 cases of patients assessed efficacy, quality of life, three-year survival, and immune function of Tianfoshen (TFS) oral liquid in treating moderate and advanced malignant tumors. The results showed that the ORR was 45.1%; the effective rate was 71.8%; 1-, 2-, and 3-year survival rates were 78.5%, 38.5%, and 10.8%, respectively; and the median survival time was 24.2 months [16].

Limitations of this study include the following: as a single-arm observational study, this study did not have a control group and also lacked a strict observation period; no ORR data (n= 492) was available because it was unable to fully study the effect of adjuvant therapy on the improvement of ORR.

Nevertheless, the large patient population included in this study may be a better representative of the actual situation of Chinese NSCLC patients.

## 5. Conclusion

In conclusion, this study demonstrated that, as an adjuvant therapy, TFS is a safe and effective therapeutic option for the Chinese NSCLC patients with Qi and Yin deficiency syndrome, who lost the opportunity for surgery, but it seems to have no effect on the ORR and DCR.

## Figures and Tables

**Table 1:** Demographic and baseline characteristics of patients.

Characteristics	Total	
	No.	%
Age (years)	56 (30-75)	
Gender		
Male	76	70.37
Female	32	29.63
Pathological diagnosis		
Adenocarcinoma	79	73.15
Squamous	23	21.3
adenosquamous	6	5.55
TMN staging		
IIIA	22	20.37
IIIB	26	24.07
IV	60	55.56
Metastatic disease		
Any	75	69.44
Lung^1^	32	29.63
Liver^1^	6	5.56
Lymph^1^	22	20.37
ECOG PS		
0	12	11.11
1	82	75.93
2	14	12.96

^1^Percentages are based on total number of patients with metastatic disease.

**Table 2:** Comparison of effective data of chemotherapy plus TFS and chemotherapy without TFS.

Treatment line	Whether to add TFS	N	Efficacy data (n, %)				ORR (%)	P(*χ*^2^)	DCR (%)	P(*χ*^2^)
			CR	PR	SD	PD				
First- line	Without TFS	42	1(2.38)	12(28.57)	15(35.71)	14(33.33)	30.95	0.756(0.097)	66.66	0.494(0.468)
	Add TFS	38	2(5.26)	11(28.95)	15(39.47)	10(26.32)	34.21		73.68	
Recurrent	Without TFS	58	0(0)	13(22.41)	23(39.66)	22(37.93)	22.41	0.624(0.241)	62.07	0.519(0.417)
	Add TFS	53	0(0)	14(26.42)	22(41.51)	17(32.08)	26.42		67.93	

**Table 3:** The improvement of TCM syndrome.

Time (Week)	Clinical control (n)	Obvious treatment effect (n)	mild treatment effect (n)	Clinical efficiency (%)
3	2	13	29	36.11
8/9	4	23	45	50.93

**Table 4:** Comparison of the TCM syndrome score at the baseline and after TFS treatment.

Category	Time (Week)	mean(SD)	Min, Max	T test(*P*)
TCM syndrome	baseline	5.15(2.20)	0, 15	—
	3	4.51(2.35)	0, 13	-14.36(<0.0001)
	8/9	3.84(2.10)	0, 13	-22.25(<0.0001)
Weight	baseline	60.59(8.79)	41, 105	—
	3	63.58(9.79)	41, 105	0.28(0.7568)
	8/9	64.25(9.25)	41, 105	3.26(0.0023)
ECOG^*∗*2^	baseline	—	—	—
	3	-0.04(0.28)	-1,1	-436.0(0.0035)
	8/9	-0.05(0.32)	-1,1	-767.5(0.0013)
FACT-L (Physiology)	baseline	18.67(4.56)	3, 26	—
	3	0.25(2.45)	-10, 17	3.25(0.0015)
	8/9	1.02(3.62)	-10, 17	7.85(<0.0001)
FACT-L (Social/Family )	baseline	17.23(3.45)	6, 27	—
	3	17.98(3.89)	6, 29	1.08(0.2645)
	8/9	18.15(3.87)	5, 28	5.56(<0.0001)
FACT-L (Emotion )	baseline	14.23(3.87)	0, 28	—
	3	14.89(3.98)	2, 24	6.12(<0.0001)
	8/9	15.23(4.33)	3, 27	9.87(<0.0001)
FACT-L (Function)	baseline	14.23(5.45)	0,28	—
	3	15.76(5.25)	0,29	6.23(<0.0001)
	8/9	16.56(5.65)	0,29	11.56(<0.0001)
FACT-L (Pain)	baseline	0.87(1.08)	0,8	—
	3	0.86(0.78)	0,6	-4.56(0.0005)
	8/9	0.87(1.21)	0,21	-4.78(<0.0001)
FACT-L (Additional attention)	baseline	22.45(5.12)	4,36	—
	3	24.56(5.12)	4,37	6.67(<0.0001)
	8/9	26.78(5.02)	4,37	10.12(<0.0001)

^2^Signed rank sum test.

TCM syndrome: traditional Chinese medicine syndrome; ECOG: Eastern Cooperative Oncology Group; FACT-L: Functional Assessment of Cancer Therapy-Lung.

**Table 5:** Frequency of adverse events associated with TFS.

Toxicity	*Grade 1(n)*	Grade 2(n)	Grade 3(n)	Grade 4(n)
Fatigue	1	0	0	0
Nausea/vomiting	2	0	1	0
diarrhea	1	0	0	0
Proteinuria	2	0	0	0
Leukopenia	1	0	0	0

## Data Availability

The data used to support the findings of this study are included within the article.
